# UMLoc: Uncertainty-Aware Map-Constrained Inertial Localization with Quantified Bounds

**DOI:** 10.3390/s26061904

**Published:** 2026-03-18

**Authors:** Mohammed S. Alharbi, Shinkyu Park

**Affiliations:** Electrical and Computer Engineering Department, King Abdullah University of Science and Technology (KAUST), Thuwal 23955, Saudi Arabia; mohammed.alharbi.3@kaust.edu.sa

**Keywords:** inertial localization, uncertainty estimation, Map-Aided Localization, quantile regression, Generative Adversarial Networks (GAN), Neural Networks

## Abstract

Inertial localization is particularly valuable in GPS-denied environments such as indoors. However, localization using only Inertial Measurement Units (IMUs) suffers from drift caused by motion-process noise and sensor biases. This paper introduces Uncertainty-aware Map-constrained Inertial Localization (UMLoc), an end-to-end framework that jointly models IMU uncertainty and map constraints to achieve drift-resilient positioning. UMLoc integrates two coupled modules: (1) a Long Short-Term Memory (LSTM) quantile regressor, which estimates the specific quantiles needed to define 68%, 90% and 95% prediction intervals serving as a measure of localization uncertainty and (2) a Conditioned Generative Adversarial Network (CGAN) with cross-attention that fuses IMU dynamic data with distance-based floor-plan maps to generate geometrically feasible trajectories. The modules are trained jointly, allowing uncertainty estimates to propagate through the CGAN during trajectory generation. UMLoc was evaluated on three datasets, including a newly collected 2-h indoor benchmark with time-aligned IMU data, ground-truth poses and floor-plan maps. Results show that the method achieves a mean drift ratio of 5.9% over a 70m travel distance and an average Absolute Trajectory Error (ATE) of 1.36m, while maintaining calibrated prediction bounds.

## 1. Introduction

Indoor localization has gained significant attention in recent years due to its wide range of applications in navigation, healthcare monitoring, emergency response and robotics [1]. Existing methods primarily depend on WiFi and Bluetooth, necessitating dense infrastructure, or on LiDAR and camera-based sensors, which require well-lit, feature-rich environments. These methods can achieve high accuracy but are costly, difficult to scale in real-world deployments [2,3], power-hungry and may interfere with human activity or raise privacy concerns [4,5,6,7]. A promising alternative is Inertial Measurement Units (IMUs). They are widely available on smartphones, require no line-of-sight, operate reliably across diverse environments and are both energy and computation-efficient.

Traditional inertial localization methods are typically divided into two categories: Strapdown Inertial Navigation Systems (SINS) and step-based approaches. SINS employ physics-based models to estimate device orientation and integrate linear acceleration to infer position [8]. Step-based methods exploit the periodic structure of human gait, relying on hand-crafted features or biomechanical models to approximate pedestrian motion through step detection, heading estimation and step length modeling [9,10]. While conceptually straightforward, these methods are highly susceptible to drift due to the accumulation of integration errors over time. Nonetheless, achieving accurate localization using only IMUs remains fundamentally challenging because of uncertainties in measurement and process, including unmodeled dynamics, sensor biases and the lack of absolute references, e.g., GPS.

In recent years, deep learning models have shown significant potential in mitigating drift in inertial positioning problems [11]. For instance, the Robust IMU Double Integration (RIDI) framework adopts a two-stage pipeline, employing a Support Vector Machine (SVM) to estimate device orientation and a Support Vector Regressor (SVR) to predict velocity vectors in the device frame [12]. Similarly, the Inertial Odometry Network (IONet), a Bidirectional Long Short-Term Memory (LSTM) model, addresses drift by estimating changes in velocity magnitude and direction from accelerometer and gyroscope data, thereby eliminating one level of integration and reducing cumulative error [13]. Extending this line of work, Robust Neural Inertial Navigation (RoNIN) introduces a robust velocity loss function. It investigates multiple network architectures, including LSTM, Temporal Convolutional Network (TCN) and ResNet, to more accurately capture ground-plane motion [14]. Nevertheless, unmodeled uncertainties can limit the model’s performance and accumulate errors, leading to trajectory drift.

Tight Learned Inertial Odometry (TLIO) pushes the boundaries of the field by utilizing a ResNet18 model and integrating it with a stochastic-cloning Extended Kalman Filter (EKF). This approach enables the simultaneous estimation of position, orientation, and sensor biases [15]. Similarly, Inertial Deep Orientation-Estimation and Localization (IDOL) combines LSTM with an EKF, enabling the model to learn velocity changes while using the filter to potentially rectify inaccurate orientation estimates [16]. Complementing this, Robust Neural Inertial Navigation Aided Visual-Inertial Odometry (RNIN-VIO) tightly couples an EKF-based VIO with a ResNet–LSTM that regresses displacements and their covariance to maintain robustness in weak-texture and motion-blurred scenes [17]. However, these methods modeled uncertainty by imposing a Gaussian distribution rather than providing calibrated prediction intervals. Although prior data-driven methods have achieved promising results, they lack explicit mechanisms to enforce spatial feasibility, often producing trajectories that intersect with obstacles, thus compromising realism and limiting practical deployment.

Despite these advances, existing inertial localization methods still suffer from inevitable drift over time. This limitation motivates exploring auxiliary information, such as map-based constraints, to provide absolute references for correcting drift and improving localization accuracy. Particle filters are widely known for integrating positional estimates with environmental constraints [18]. These filters propagate multiple hypotheses of location based on odometry, retaining or discarding particles based on their consistency with the map and sensor observations. While they are effective in practical applications [19,20], particle filters are highly sensitive to odometry noise, leading to premature removal of valid hypotheses. To address this issue, ref. [21] introduced learnable map embeddings that adaptively reweight particle distributions, enhancing robustness against noisy motion estimates. Yet, this method cannot propagate map consistency violations back into the feature learning process, hence limiting further improvements in localization accuracy.

The lack of an accurate inertial localization model that explicitly incorporates the spatial constraints and uncertainty bounds into position estimation motivates the design of an end-to-end localization framework to address these limitations. In this work, we introduce UMLoc, an Uncertainty-aware Map-constrained inertial Localization framework that aims to generate spatially feasible trajectories with quantified bounds. Thus, it elevates the task from unconstrained inertial tracking to map-compliant localization with probabilistic guarantees. The proposed architecture consists of two main components: (1) a quantile regression module based on an LSTM network, which estimates prediction intervals for velocity uncertainty; and (2) a Conditional Generative Adversarial Network (CGAN) with a cross-attention mechanism, which unifies quantile-derived uncertainty and distance-based map feasibility for trajectory generation. The key innovation lies in its structured conditioning via a cross-attention mechanism. It enables the model to simultaneously generate spatially consistent pedestrian positions within a global reference frame while providing a confidence measure that is both robust and interpretable. To achieve our objectives, curriculum learning is used to optimize the multitasking framework, starting with an adapted pinball loss to obtain predictive bounds. Then, an adversarial loss and a supervised trajectory loss with a map-compliance penalty are used to train the CGAN for map-compliant trajectory prediction. The proposed UMLoc is extensively evaluated across three datasets, our own dataset and two publicly available datasets. Furthermore, UMLoc is compared against strong baselines, including RoNIN-TCN, RoNIN Bi-LSTM [14] and RNIN [17], demonstrating superior accuracy, robustness and generalization. The main contributions of this work are as follows:

- We introduce an LSTM-based quantile prediction module that estimates prediction intervals for velocity, which are integrated to form a positional bound for position estimates.
- We design a CGAN with a cross-attention mechanism to generate trajectories that are consistent with both the learned uncertainty bounds and a 2D environmental map, thereby ensuring map-compliant localization.
- We couple these two modules into a novel end-to-end learning framework for inertial localization that: (1) Effectively propagates IMU uncertainty through to the final trajectories; (2) Yields reliable, drift-reduced position predictions.

Beyond these technical contributions, we also establish an indoor localization benchmark dataset consisting of synchronized IMU data, ground-truth poses and floor plan maps.

This paper is organized as follows: Section 2 describes the formulation of the localization problem. Section 3 introduces the proposed UMLoc with its data preparation and training process. Then, the inertial localization results and the ablation study are presented in Section 4. Finally, concluding remarks on the proposed methods are presented in Section 5.

## 2. Problem Description

We formulate the inertial localization problem as a learning task. It exploits IMU data from a handheld device (e.g., a smartphone) and a corresponding 2D map of the environment to predict the pedestrian’s position. Let $X_{1:t}=\left(x_{1},\ldots ,x_{t}\right)$ denote IMU measurements from the initial time to the current time *t*, where each vector $x_{\tau }={\left(a_{x,\tau },a_{y,\tau },a_{z,\tau },\omega_{x,\tau },\omega_{y,\tau },\omega_{z,\tau }\right)}^{⊤}\in R^{6}$ at time τ∈{1,…,t} contains three-axis linear acceleration $\left(a_{x,\tau },a_{y,\tau },a_{z,\tau }\right)$ and three-axis angular velocity $\left(\omega_{x,\tau },\omega_{y,\tau },\omega_{z,\tau }\right)$.

The goal is to estimate the corresponding sequence of positions $P_{1:t}=\left(p_{1},\ldots ,p_{t}\right)$ within a time window while accounting for drift and spatial constraints. To facilitate this, we define the velocity sequence as $V_{1:t}=\left(v_{1},\ldots ,v_{t}\right)$, where each velocity vector $v_{\tau }={\left(v_{x,\tau },v_{y,\tau }\right)}^{⊤}\in R^{2}$ represents motion in the 2D plane. The corresponding position at time τ is denoted by $p_{\tau }=\left(p_{x,\tau },p_{y,\tau }\right)$, lying in a global 2D coordinate frame that is aligned with the environmental map M. We assume that the vertical position $p_{z,\tau }$ remains constant and that the map M is known a priori. To address the problem, we decompose the localization task into two interconnected sub-tasks.

### 2.1. Uncertainty Estimation via Quantile Regression

Let $v_{t}$ denote a random variable representing the ground-truth velocity at time *t*. For each time step *t*, the probability that $v_{t}$ falls within the conditional lower and upper quantiles, given IMU observation $X_{1:t}$, is defined as

(1) $$P\left[q_{t}^{L}⩽v_{t}⩽q_{t}^{U}\;|\;v_{1},X_{1:t}\right]=1-2\alpha ,$$

where the inequality in (1) is applied element-wise, and $q_{t}^{L},q_{t}^{U}\in R^{2}$ represent the conditional lower and upper quantiles of $v_{t}$ conditioned on the initial velocity $v_{1}$ and IMU data $X_{1:t}$, with α∈(0,0.5) denoting the tail probability. These quantiles define the prediction interval at confidence level 1−2α, expressed as $\left[q_{t}^{L},q_{t}^{U}\right]$.

To estimate these quantiles, we train a quantile regression network:

(2) $$\left({\hat{q}}_{t}^{L},\;{\hat{q}}_{t}^{U}\right)=F_{q}\left(v_{1},X_{1:t};\theta_{q}\right),$$

where $F_{q}$ denotes a quantile regression network parameterized by $\theta_{q}$, trained using the adapted pinball loss described in Section 3.2. The predicted quantiles define a prediction interval $\left[{\hat{q}}_{t}^{L},{\hat{q}}_{t}^{U}\right]$ such that $\hat{P}\left[{\hat{q}}_{t}^{L}⩽v_{t}⩽{\hat{q}}_{t}^{U}\right]=1-2\alpha$. These quantiles capture both model uncertainty and intrinsic data variability, serving as constraints for subsequent trajectory generation. By providing statistically grounded uncertainty margins, the learned bounds improve the reliability and robustness of inertial localization.

### 2.2. Map-Constrained Trajectory Generation

A generative neural network model is designed to generate realistic velocity trajectories by sampling from the learned distribution $p_{G}$:

(3) $${\hat{v}}_{t}∼p_{G}\left({\hat{v}}_{t}|y_{t}\right),\;y_{t}:=\left\{I_{1:t},M,{\hat{q}}_{t}^{L},{\hat{q}}_{t}^{U}\right\},$$

where $y_{t}$ is the condition, $I_{1:t}$ represents IMU features from the initial time to current time *t* and M encodes the environmental map. The prediction interval ${\hat{q}}_{t}^{L}$ and ${\hat{q}}_{t}^{U}$ act as quantile-based quantified uncertainty bounds that guide trajectory generation. Subsequently, multihead cross-attention is employed to fuse the IMU dynamics and distance-based map feasibility.

## 3. Uncertainty-Aware Map-Constrained Inertial Localization

**Figure 1:**
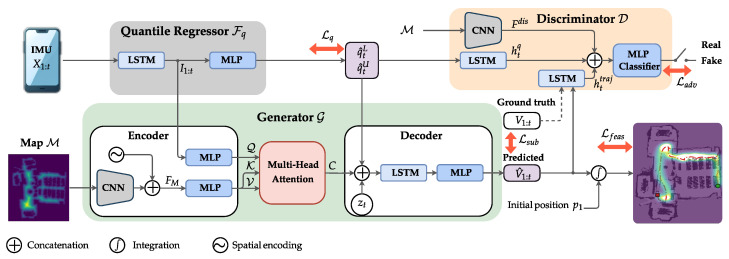
Schematic of UMLoc. IMU sequences $X_{1:t}$ feed an LSTM quantile regressor that predicts lower and upper conditional quantiles, while CNN encodes the distance map M. Cross-attention then fuses the IMU with the map features, and the decoder generates velocities ${\hat{V}}_{1:t}$, which are integrated to obtain the positions. The discriminator D uses a CNN encoder and 2 LSTM encoders, followed by MLP, to distinguish between real and generated sequences.

UMLoc is a two-stage framework that (1) estimates predictive uncertainty bounds using an LSTM-based quantile regression module and (2) generates spatially feasible velocity trajectories via a CGAN conditioned on an environmental map. Figure 1 illustrates the end-to-end pipeline of the proposed UMLoc framework.

We begin by introducing the quantile prediction module, followed by the map-constrained CGAN, which employs a cross-attention mechanism to generate trajectories consistent with both the predicted uncertainty intervals and the environmental map. We then describe the data-collection process used for training and present the progressive training curriculum designed to optimize the model end-to-end.

### 3.1. LSTM-Based Quantile Regression Module

Localization drift primarily stems from propagated modeling uncertainties, IMU sensor biases and variability in human motion patterns. Instead of relying on deterministic velocity estimates, as in prior work [13,14,16], we employed a quantile regression approach to estimate prediction intervals, providing interpretable measures of uncertainty.

Formally, given an IMU sequence $X_{1:t}$, the quantile regression module computes the lower and upper quantiles, ${\hat{q}}_{t}^{L}=\left({\hat{q}}_{x,t}^{L},{\hat{q}}_{y,t}^{L}\right)$ and ${\hat{q}}_{t}^{U}=\left({\hat{q}}_{x,t}^{U},{\hat{q}}_{y,t}^{U}\right)$, of the ground-truth $v_{t}=\left(v_{x,t},v_{y,t}\right)\in R^{2}$. It is conditioned on the initial velocity $v_{1}$ and the IMU data $x_{1},\ldots ,x_{t}$, at each time *t*. To compute these quantiles, the module is trained using an adapted pinball loss. The loss extends the classic formulation [22] by the cumulative sum of the predicted velocity quantiles to estimate positional displacement over the interval [1,T]:

(4) $$\begin{array}{cc} L_{q} & =\frac{1}{4T}\sum_{t=1}^{T}\left[\rho_{\alpha }\left(\sum_{k=1}^{t}\left(v_{x,k}-{\hat{q}}_{x,k}^{L}\right)\right)+\rho_{1-\alpha }\left(\sum_{k=1}^{t}\left(v_{x,k}-{\hat{q}}_{x,k}^{U}\right)\right)\right]\\ \; & \;+\frac{1}{4T}\sum_{t=1}^{T}\left[\rho_{\alpha }\left(\sum_{k=1}^{t}\left(v_{y,k}-{\hat{q}}_{y,k}^{L}\right)\right)+\rho_{1-\alpha }\left(\sum_{k=1}^{t}\left(v_{y,k}-{\hat{q}}_{y,k}^{U}\right)\right)\right], \end{array}$$

where *T* is the trajectory length, and $\rho_{\alpha }\left(u\right)=\max\left(\alpha u,\left(\alpha -1\right)u\right)$.

In inertial navigation, positions are obtained by integrating velocities; thus, the dominant failure mode is drift, where small systematic velocity errors accumulate over time. To align the training objective with this property, we apply the pinball loss to the cumulative velocity residuals. Therefore, minimizing (4) encourages the predicted quantiles to provide calibrated bounds not only on instantaneous velocity but also on the integrated displacement, which directly governs long-horizon position error.

We stacked two layers of unidirectional LSTM layers with 64 units each to extract sequential features $I_{1:t}=\mathrm{LSTM}\left(x_{1:t}\right)$. The resulting features are passed through two linear layers with shared weights of size (20,4), producing four scalar outputs at each time step. These scalars correspond to the predicted lower and upper quantiles for the two velocity components, $v_{x,t}$ and $v_{y,t}$. Together, these components constitute the quantile regressor $F_{q}$ shown in Figure 1. In this work, we experimented with different values of α=(0.16,0.05,0.025), corresponding to (68%,90%,95%) prediction intervals, respectively. The resulting interval $\left({\hat{q}}_{t}^{L},{\hat{q}}_{t}^{U}\right)$ serves as an uncertainty-aware constraint, which conditions the trajectory generation module in the second stage of UMLoc.

### 3.2. Conditional Generative Adversarial Network (CGAN)

The CGAN module consists of two components: a generator network G and a discriminator network D, as illustrated in Figure 1. The generator G learns to produce realistic velocity trajectories by sampling from the learned distribution $p_{G}$ as in (3). The discriminator D learns to classify real and generated velocity trajectories. The overall CGAN objective integrates an adversarial loss and a supervised estimation loss with a map feasibility penalty to jointly improve localization accuracy, spatial feasibility and drift resilience.

The generator G architecture is shown in Figure 1 and consists of a CNN–attention encoder and an LSTM-based decoder. The encoder processes the 2D environmental map $M\in R^{H\times W}$ to generate a feature map. We encode this map using a lightweight CNN consisting of two blocks (32 and 64 channels) of Convolution, batch normalization and Rectified Linear Unit (ReLU), each followed by 2×2 max-pooling. Then a depthwise 3×3 convolution and a final 1×1 pointwise convolution that projects features to the context feature dimension. Then, it is augmented by the 2D coordinates (spatial encoding) to provide spatial awareness as $F_{M}\in R^{H_{r}\times W_{r}\times \left(64+2\right)}$.

The feature map $F_{M}$ is reshaped into a sequence of $F=H_{r}W_{r}$ tokens and linearly projected by the MLP to obtain the keys and values $K,V\in R^{F\times d}$. In parallel, the temporal IMU features $I_{1:t}$ are projected to the same attention dimension *d* via an MLP to form the temporal queries $Q\in R^{T\times d}$. Then, we apply multi-head cross-attention with h=4 heads, where each head attends from the temporal queries to the spatial tokens. The head outputs are concatenated and projected to produce the fused context sequence $C\in R^{T\times d}$ as follows:

$$\begin{array}{cc} C & =Concat\left[head_{1},\ldots ,head_{h}\right]W^{o},\\ head_{i} & =\mathrm{softmax}\left(\frac{\left(QW_{i}^{q}\right){\left(KW_{i}^{k}\right)}^{⊤}}{\sqrt{d_{h}}}\right)\left(VW_{i}^{v}\right),\;d_{h}=d/h \end{array}$$

where $W_{i}^{q}$, $W_{i}^{k}$, $W_{i}^{v}$ and $W^{o}$ are learnable projection matrices. The resulting context vector aggregates map information relevant to the current IMU state, allowing the model to selectively emphasize spatial regions (e.g., high-clearance corridors vs. near-obstacle areas) when generating the trajectory.

Overall, this design grounds the IMU sequence in the environment’s spatial context while enforcing feasibility constraints that prevent information leakage from invalid areas. To introduce controlled stochasticity and enhance diversity, the cross-attention context vector $c_{t}$ at time step *t* is concatenated with latent Gaussian noise $z_{t}$∼N(0,I). At each step, we form the decoder input by concatenating the cross-attention context $c_{t}$, the latent Gaussian noise and embedded uncertainty bounds $\phi ({\hat{q}}_{t}^{L},{\hat{q}}_{t}^{U})$. The decoder is a recurrent predictor parametrized by $\theta_{dec}$ comprising a single-layer LSTM with 64 units. The LSTM hidden state is then mapped through a linear layer to generate the velocity estimate ${\hat{v}}_{t}$ conditioned on the predicted quantiles $\left({\hat{q}}_{t}^{L},{\hat{q}}_{t}^{U}\right)$ and cross-attention context vector, ensuring that the generated trajectories remain within the learned quantile-based uncertainty bounds as follows:

(5) $${\hat{v}}_{t}=F_{dec}\left(Concat\left[z_{t},c_{t},\phi \left({\hat{q}}_{t}^{L},{\hat{q}}_{t}^{U}\right)\right];\theta_{dec}\right).$$

Thus, the positions follow discrete integration: ${\hat{p}}_{t}={\hat{p}}_{t-1}+\Delta t\;{\hat{v}}_{t}$, where Δt is the sampling period which corresponds to 0.0167s in our experiments.

To differentiate between real velocity trajectory $V_{1:t}$ and generated trajectory ${\hat{V}}_{1:t}$, the discriminator D is conditioned on $\left\{M,{\hat{q}}_{t}^{L},{\hat{q}}_{t}^{U}\right\}$. We used 2 LSTM encoders with 32 units to process the trajectory and the corresponding quantile bounds, respectively, and use their final hidden states, denoted by $h_{t}^{traj}$ and $h_{t}^{q}$. In addition, a CNN extracts a feature vector $F^{dis}$ from the 2D map M. The MLP classifier then takes the concatenation of these features and hidden states to distinguish between real and generated velocity trajectories.

(6) $$\hat{L}=MLP\left(Concat\left[h_{t}^{traj},h_{t}^{q},F^{dis}\right]\right),$$

where $\hat{L}$ denotes the discriminator’s predicted label, indicating whether the selected trajectory sample corresponds to a ground truth (real) or generated (fake) data.

Empirically, adding $I_{1:t}$ to D reduced stability and did not improve the framework’s performance. The discriminator D is trained to minimize the following adversarial loss $L_{D}$:

(7) $$L_{D}=-E_{{\hat{v}}_{t}∼p_{G}\left({\hat{v}}_{t}|y_{t}\right)}\left[\log\left(1-D\left({\hat{v}}_{t}|y_{t}\right)\right)\right]-E_{v∼\mathrm{real}}\left[\log\left(D\left(v_{t}|y_{t}\right)\right)\right].$$

We train the generator to encourage the production of trajectories with low final displacement error (FDE). Specifically, we generate K=20 samples and select the trajectory with the lowest FDE. The generator G is optimized with a composite weighted objective that combines adversarial loss and a supervised velocity and position loss with a map feasibility penalty, $L_{G}=L_{adv}+\lambda_{feas}\;L_{feas}+\lambda_{sup}\;L_{sup}$, where

$$\begin{array}{cc} L_{adv} & =-E_{{\hat{v}}_{t}∼p_{G}\left({\hat{v}}_{t}|y_{t}\right)}\left[\log D\left({\hat{v}}_{t}|y_{t}\right)\right],\\ L_{feas} & =\frac{1}{T}\sum_{t=1}^{T}{\left(\max(R_{s}-M\left({\hat{p}}_{t}\right),0)\right)}^{2},\\ L_{sup} & =\frac{1}{T}\sum_{t=1}^{T}\left[\gamma ∥p_{t}-{\hat{p}}_{t}{∥}_{2}^{2}+\left(1-\gamma \right){∥v_{t}-{\hat{v}}_{t}∥}_{2}^{2}\right]. \end{array}$$

Here, $L_{feas}$ penalizes trajectories that are close to or intersect with obstacles in the map. This penalty uses a safety margin, $R_{s}$, which is applied when the trajectories are within $R_{s}$ distance of any obstacles. $R_{s}$ is chosen empirically to be 0.4m. The supervised loss $L_{sup}$ enforces supervision, with γ=0.3 chosen to balance the velocity and position contribution. The weights are empirically chosen to balance each learning task and stabilize training, with $\lambda_{sup}=5$ and $\lambda_{feas}$ linearly ramped over training iterations *i* to gradually enforce spatial feasibility $\lambda_{feas}=\min\left(0.5,\max\left(0,0.5\frac{i-10000}{2000}\right)\right)$.

### 3.3. Data Preparation for Training and Evaluation

Each data sample comprises 6-DoF IMU readings collected from an Android smartphone (Samsung Galaxy S23 Ultra, Vietnam) placed in the pedestrian’s pants pocket. It consists of 3-axis linear accelerations and 3-axis angular velocities, in addition to ground-truth position data and a 2D map generated using the SLAM API of the Stereolabs (Paris, France) ZED 2i camera system, which is fixed to the pedestrian’s chest. The IMU measurements are reported in the device body frame {b}, which changes dynamically as the device moves. In contrast, both the ground-truth positions and the 2D map are represented in a fixed global frame {g}. It is defined by a right-handed *z*-up coordinate system with the *z*-axis aligned with the gravity. Hence, the Android Game Rotation Vector (GRV) [23] is used to estimate the smartphone’s orientation $Q_{t}$ in quaternion form. It is then applied to transform the IMU measurement $x_{t}$ from the body frame {b} to the global frame {g}: $x_{t}^{g}=Q_{t}\;x_{t}^{b}\;Q_{t}^{*},$ where $Q_{t}^{*}$ denotes the quaternion conjugate of $Q_{t}$.

During training, both IMU data and ground-truth velocities are randomly rotated by an angle ψ∈[0,2π) on the horizontal plane [14]. This augmentation compels the model to learn heading-invariant motion patterns such as steps, turns and lateral movements. As a result, the trajectory direction naturally emerges from successive velocity predictions, leading to better generalization at inference. During testing, the GRV is used to estimate the smartphone’s orientation to ensure that the *z*-axis of the output frame remains aligned with gravity.

In addition, a 2D map is constructed as a binary occupancy grid $M_{occ}\in {\left\{0,1\right\}}^{H\times W}$, where static structures such as walls, doors and offices are represented as obstacles. Hence, $M_{occ}\left(i,j\right)$ takes the value 1 if cell (i,j) is free and 0 otherwise. To enable differentiability and capture rich spatial context, a Euclidean distance transform $M_{EDT}$ is applied to the occupancy grid, producing a smoothed representation. In $M_{EDT}$, each cell stores the shortest Euclidean distance to the nearest obstacle.

The resulting distance values are converted to metric units using the map resolution *r* (in meters per pixel), yielding the final map representation $M\left(i,j\right)=r\;M_{EDT}\left(i,j\right)$. This continuous map-aware encoding promotes numerical stability, embeds spatial proximity to obstacles and supports gradient-based learning.

### 3.4. Training Process

We adopted a curriculum-based learning strategy to meet our objectives of predictive bounds, feasible trajectory generation and reduced drift in inertial localization. The training is conducted in three successive phases to stabilize adversarial learning and propagate uncertainty from quantile bounds into the map-conditioned generator.

First, the quantile module was pretrained for 150 epochs by minimizing $L_{q}$. Next, CGAN was trained for 50,000 iterations to generate global velocity sequences, while optimizing $L_{G}$ with the quantile module frozen, starting with a warm-up supervised learning to minimize $L_{sup}$. After that, we annealed the adversarial term $L_{adv}$ to shape realistic and multimodal motion once predictions were reasonable. Then, we ramped the feasibility loss $L_{feas}$ to enforce map compliance. Finally, both modules were fine-tuned jointly in an end-to-end manner.

We implemented the proposed model using PyTorch 2.1 and ran it on an NVIDIA RTX 2080 Ti GPU (12 GB). In this work, we tuned the hyperparameters using optuna [24] for hyperparameter optimization. For training, the Adam optimizer was used, with learning rates of (0.001,0.0001,0.0002) for the quantile module, generator and discriminator, respectively. We used the two-time-scale update rule [25], training the discriminator with a larger learning rate and multiple updates per generator step, which empirically stabilizes CGAN training and improves convergence. A batch size of 16 and a window of size 120, corresponding to 2s, is employed to train the proposed model. A scheduler is used to adjust the learning rate of the quantile module, reducing it by a factor of 0.75 if the validation loss does not decrease for 15 epochs.

The end-to-end model required approximately 14 h of training. Regarding the inference time, UMLoc requires an average of 363ms per full trajectory. For streaming operations, the average latency is 4.5ms per prediction window, using K=20 CGAN samples per instance. It corresponds to an effective inference rate of approximately 200 Hz, which supports real-time deployment at 60–100 Hz (i.e., 16.7–10ms budget per step), providing a 3.7–2.2× computational margin, given access to the 2D map.

## 4. Results

### 4.1. Experimental Setup

#### 4.1.1. Datasets

We evaluated the proposed UMLoc model on three inertial localization datasets to assess its performance, generalization and robustness. **RoNIN** [14] and **RNIN** [17] are two publicly available datasets for inertial localization (see Table 1). To complement these benchmarks, we introduce a new dataset comprising over 2 h of indoor pedestrian trajectory data collected across 5 buildings with 12 different map layouts. We recorded IMU and camera data at a sampling rate of 60 Hz, with each trajectory lasting no longer than 10 min. We developed a custom Android application to log all sensor streams. This dataset establishes a new benchmark for map-aware inertial localization in indoor environments.

#### 4.1.2. Baselines and Metrics Definitions

To evaluate the proposed inertial localization model UMLoc, we compared its performance against three state-of-the-art baselines:

- **RoNIN LSTM** [14]: A recurrent model that employs LSTM layers with bilinear layers to regress velocity directly from IMU data.
- **RoNIN TCN** [14]: A convolution-based alternative that utilizes a Temporal Convolutional Network (TCN) for velocity prediction from IMU data.
- **RNIN-VIO** [17]: An uncertainty-aware deep learning model that explicitly predicts displacement along with its associated covariance from IMU data.

By benchmarking against diverse architectures and datasets, we aim to comprehensively demonstrate the effectiveness and robustness of UMLoc across different environments, people, devices and multiple levels of sensor noise captured in the datasets.

For systematic evaluation, we employ multiple quantitative metrics for each position trajectory of length *T*:

- **Absolute Trajectory Error (ATE):** The Root Mean Square Error (RMSE) between estimated and ground-truth positions is given as $\mathrm{ATE}=\sqrt{\frac{1}{T}\sum_{t=1}^{T}\left|\right|{\hat{p}}_{t}-p_{t}{\left|\right|}_{2}^{2}}$. It reflects global trajectory consistency and accumulates over time due to drift.
- **Relative Trajectory Error (RTE):** The RMSE of position differences over a fixed time interval (Δt=1 min) is calculated as
  $$\mathrm{RTE}=\sqrt{\frac{1}{T-\Delta t}\sum_{t=1}^{T-\Delta t}\left|\right|\left(p_{t+\Delta t}-p_{t}\right)-\left({\hat{p}}_{t+\Delta t}-{\hat{p}}_{t}\right){\left|\right|}_{2}^{2}}.$$
  It captures local trajectory consistency.
- **FDE:** The final displacement error is normalized by the total trajectory distance *L*, $FDE={∥{\hat{p}}_{T}-p_{T}∥}_{2}^{2}/L$. It quantifies long-term drift relative to the path distance.

#### 4.1.3. Smartphone Orientation Handling

RoNIN performs pre-alignment of the tracking and IMU devices at the start and end of each recording session [14]. Specifically, it employs two smartphones, one dedicated to collecting ground-truth trajectories and the other to capturing the pedestrian’s actual motion data. In addition, orientation supervision is applied during training using ground-truth device orientations. However, at test time, the model relies on device orientation, making orientation errors a dominant source of drift. RNIN, by contrast, employs controlled setups (e.g., VICON motion capture systems or rigid camera–IMU assemblies) and leverages ground-truth orientations, thereby bypassing orientation noise inherent in free-hand use. However, in real-world scenarios, ground-truth orientations are unavailable. To address this, UMLoc is trained and evaluated exclusively using GRV, which reflects realistic deployment conditions. This design makes our experiments particularly challenging, as no ground-truth orientations are assumed.

### 4.2. Experiment Results

For each dataset (RoNIN, RNIN, ours), we adopted a zero-shot protocol by holding out entire sequences from unseen users, devices and buildings for testing. The zero-shot testing demonstrates performance and generalization to unseen data. Also, in all experiments, we used the 95% prediction interval for the UMLoc model, as it provided more robust and accurate localization than the 68% and 90% intervals, unless stated otherwise. Table 2 reports the evaluation metrics for all the datasets on UMLoc against baseline models to summarize their performance. Across these splits, UMLoc transfers without fine-tuning, maintaining competitive accuracy on new people and phones (a capability particularly demonstrated by the results on the RoNIN and RNIN datasets). Furthermore, cross-building testing in our dataset demonstrates that UMLoc maintains competitive accuracy in diverse layouts.

#### 4.2.1. Evaluation on RoNIN and RNIN Datasets

RoNIN and RNIN are two widely used indoor inertial benchmark datasets containing diverse pedestrian trajectories [14,17]. These datasets are collected from different devices and by different people. The RoNIN dataset comprises over 40 h of recordings and uses a 85/15 train–test split, whereas RNIN includes 7 h of data with a 90/10 split, following the original papers’ setup. Since neither dataset provides map information, we supplied UMLoc’s CNN branch with a *uniform feasibility map* to maintain identical model capacity and ensure a fair comparison that quantifies IMU-only performance.

Table 2 shows that UMLoc without map conditioning achieves an ATE of 6.58m and FDE of 3.5% over the traveled distance on the RoNIN dataset. For RNIN, it attains ATE of 1.47m and RTE around 2m over a period of 1min. These results show marginal improvements across all metrics relative to the strongest baselines (see the ”Improvement” rows).

#### 4.2.2. Evaluation on Our Dataset

**Figure 2:**
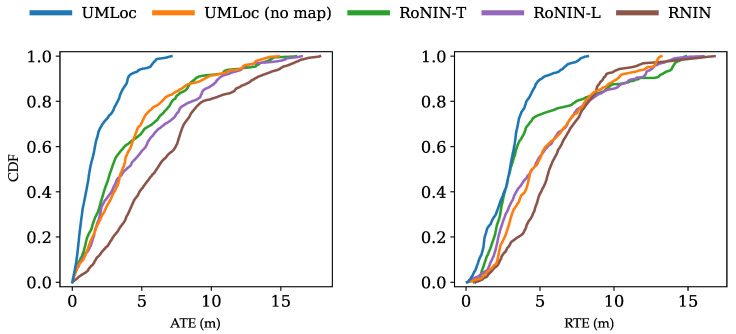
CDFs of ATE (**left**) and RTE (**right**) on our datasets’ unseen testing split. UMLoc achieves 80% cumulative probability below 2.5m error, whereas other models have it at 7.5m.

**Figure 3:**
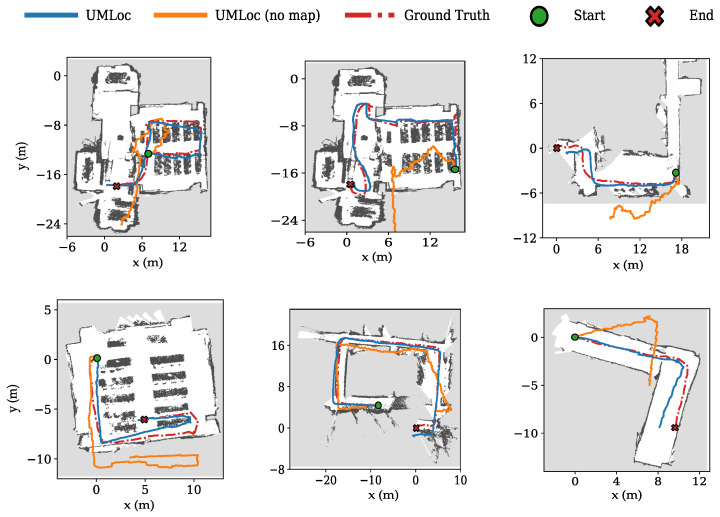
Selected 2D trajectory visualizations. We selected 6 trajectories from our dataset that include the map.

We split the trajectories into an 85/15 split for training and testing. The testing split includes a mix of unseen layouts and trajectories. All baseline models were retrained using identical splits to ensure a fair comparison. On our dataset, UMLoc with map conditioning outperforms all baselines, as well as its IMU-only variant, achieving over 50% improvement over the strongest baseline. UMLoc achieved an average drift of 5.9% over an average travel distance of 70m, corresponding to a total combined travel distance over 400m for testing trajectories (see Table 2). Also, the cumulative distribution functions (CDFs) of ATE and RTE show that UMLoc is the first to reach its maximum error, as illustrated in Figure 2. Figure 3 provides a visualization of selected unseen trajectories on their maps to compare the UMLoc with its IMU-only variant. The UMLoc accurately predicts map-compliant trajectories and effectively avoids obstacle areas.

**Figure 4:**
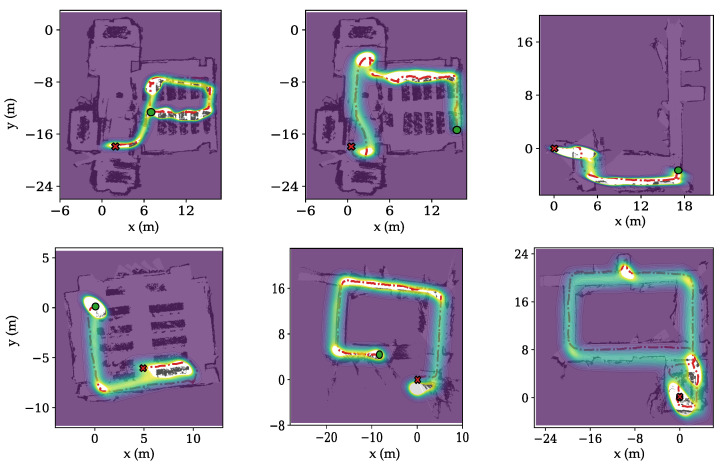
Illustrations of 20 trajectory samples from CGAN for 6 testing trajectories from our dataset. High intensity represents a higher probability of pedestrian location.

To test the model’s ability to generate multiple trajectories that follow the map constraints and to assess its uncertainty estimation, we draw 20 trajectory samples from the CGAN. In Figure 4, the density is demonstrated over the 2D map with the ground truth trajectory. The resulting high-density ridge closely follows the ground-truth path while remaining within free space, indicating slight bias and good adherence to map constraints.

#### 4.2.3. Robustness Test

To evaluate the resilience of the proposed UMLoc pipeline under realistic operating conditions, we introduced two common sensor degradations *only at the testing time*:

*(i)* **Additive Gaussian noise.** Each accelerometer and gyroscope reading was corrupted with zero-mean and standard deviations $\sigma \in \{0,\;0.1\sigma_{IMU},\;0.5\sigma_{IMU},\;1.0\sigma_{IMU},\;5.0\sigma_{IMU}\}$, where $\sigma_{IMU}$ is the captured IMU standard deviation. This perturbation mimics thermal noise in low-cost MEMS units, human-induced vibrations, electromagnetic interference and the gradual increase in noise power caused by sensor aging.

*(ii)* **Random sample dropout.** A total of 10% of IMU frames were randomly replaced with 0. This scenario reflects real-world issues such as packet loss on wireless links and power-saving modes.

For the above two settings, we computed the Prediction-Interval Coverage Probability (PICP) and Average Interval Width (AIW) to evaluate the quantile calibration:

(8) $$\begin{array}{cc} \mathrm{PICP}\; & =\frac{1}{T}\sum_{t=1}^{T}1\;\left[{\hat{q}}_{t}^{L}\leq v_{t}\leq {\hat{q}}_{t}^{U}\right], \end{array}$$

(9) $$\begin{array}{cc} \mathrm{AIW}\; & =\frac{1}{T}\sum_{t=1}^{T}{∥{\hat{q}}_{t}^{U}-{\hat{q}}_{t}^{L}∥}_{2}, \end{array}$$

where the inequality in (8) is applied element-wise. Since RoNIN does not natively provide uncertainty measures for a fair comparison, we used RNIN as the baseline for the robustness test. We derived the component-wise prediction intervals of 68%, 90% and 95% (corresponding to 1−2α) by using multiples of the predicted standard deviation ${\hat{\sigma }}_{t}$, specifically $(1{\hat{\sigma }}_{t}$, $1.64{\hat{\sigma }}_{t}$ and $2{\hat{\sigma }}_{t})$ (detailed in Appendix A). For example, the 95% interval bounds were calculated as

(10) $${\hat{q}}_{t}^{L}={\hat{v}}_{t}-\;2{\hat{\sigma }}_{t},\;{\hat{q}}_{t}^{U}={\hat{v}}_{t}+\;2{\hat{\sigma }}_{t}.$$

**Figure 5:**
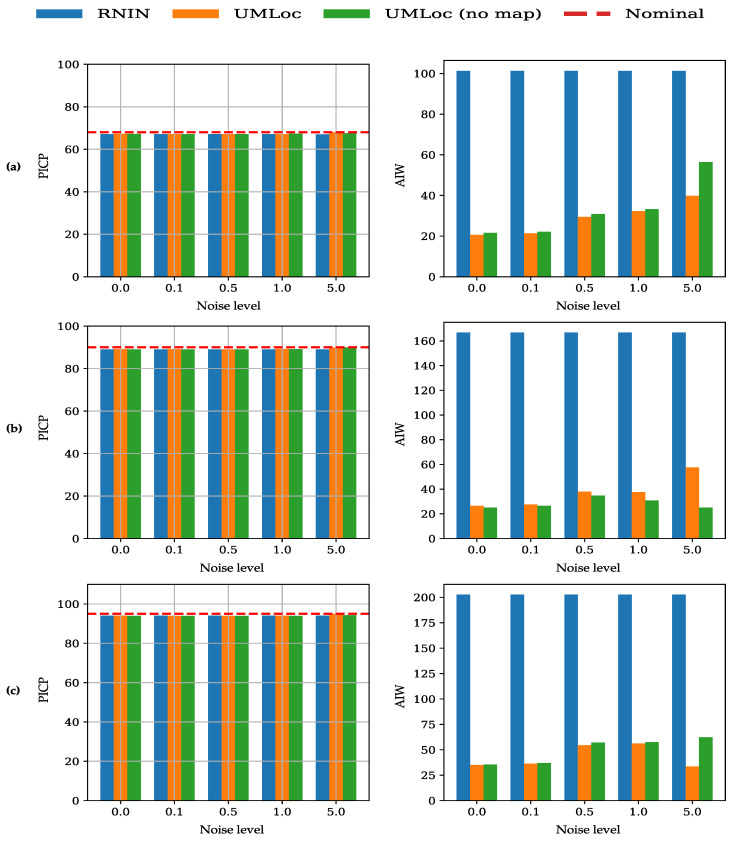
Robustness test of three models under increasing IMU noise and 10% random dropout. On the left is the PICP, and AIW is shown on the right, such that (**a**) represents the 68% prediction interval; (**b**) represents the 90% prediction interval; (**c**) represents the 95% prediction interval. All three models maintain PICP near the nominal level across all noise levels. However, our proposed model, UMLoc, with and without map variants, adaptively widens their prediction interval as the noise level increases. In contrast, RNIN gives a high constant AIW, indicating that PICP maintained close to the nominal level reflecting good coverage. Hence, the plots highlight the superior calibration of the proposed UMLoc model.

The evaluation of the prediction interval is shown in Figure 5, which reports PICP and AIW for the 3 intervals {68%,90%,95%}. The figure highlights how well the intervals are maintained against different operating conditions. UMLoc consistently maintains its coverage probability close to the nominal target and exhibits the most adaptive increase in interval width, even at high noise levels. In contrast, RNIN maintains coverage, but the intervals are unrealistically wide. Overall, UMLoc provides the best balance, effectively maintaining high coverage while keeping a reasonable interval width under noisy conditions and clearly outperforms RNIN in terms of robustness and calibration. These results demonstrate that our method’s explicit uncertainty quantification is robust against the inevitable noise, interference and data gaps encountered in practical deployments.

#### 4.2.4. Map Sensitivity Test

We further expand the quantitative performance analysis by conducting two inference-time sensitivity tests that perturb the map M while keeping the IMU stream and the ground-truth trajectory fixed. The resulting degradation is reported in ATE. First, we inject **Gaussian map noise** to emulate map extraction and discretization errors:

(11) $$\tilde{M}=\max\left\{0,\;M+\epsilon \right\},\;\epsilon ∼N\left(0,\sigma_{m}^{2}\right),$$

where $\sigma_{m}$ is specified in meters. Second, we evaluate **map-trajectory misregistration** by applying a rigid 2D transformation to the distance map only:

(12) $$\tilde{M}=M\;\left(R^{-1}\left(p-t\right)\right),$$

where *R* is a planar rotation by θ degrees, and t is a translation (converted from meters to pixels using the map resolution).

**Figure 6:**
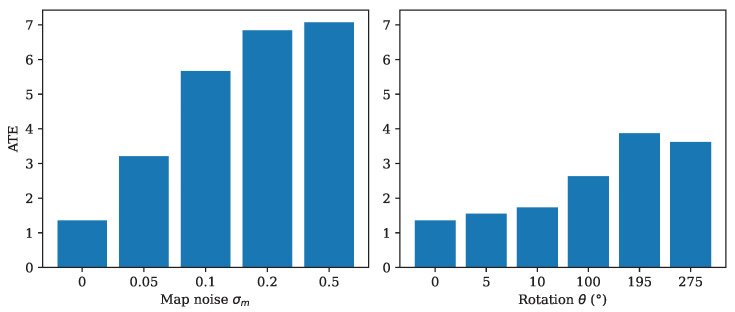
Sensitivity analysis of the proposed method under map perturbations. **Left**: Effect of additive Gaussian noise on localization accuracy. **Right**: Effect of map rotation by an angle $\theta {(}^{\circ })$ on ATE. The results indicate a gradual performance degradation as map noise and misalignment increase, while the method remains relatively robust to small rotational errors.

Figure 6 presents the ATE as a function of map noise and misalignment. The results illustrate performance degradation and clearly delineate UMLoc’s sensitivity boundaries with respect to map quality and map trajectory misregistration.

#### 4.2.5. Ablation Study

We conducted an ablation study to explicitly evaluate the contribution of key components of UMLoc, focusing on *(i)* multi-head cross-attention, *(ii)* generator loss $L_{G}$ and *(iii)* the impact of map conditioning. Table 3 reports the ATE results of the ablation study on our dataset.

*(i)* **Multi-head cross-attention.** To evaluate the effectiveness of the cross-attention module, we replaced it with a simple MLP that takes the concatenated IMU feature $I_{1:t}$ and map feature $F_{M}$ as input. With this modification, ATE increases compared to the proposed model with the cross-attention module. This confirms that multi-head cross-attention more effectively fuses IMU and map information, leading to improved localization performance.

*(ii)* **Generator loss $L_{G}$.** To evaluate the contribution of each component in the generator loss, we removed individual terms one at a time. Specifically, we ablated the feasibility loss $L_{feas}$, the position term $∥p_{t}-{\hat{p}}_{t}{∥}_{2}^{2}$ in the supervised loss and the velocity term $∥v_{t}-{\hat{v}}_{t}{∥}_{2}^{2}$ in the supervised loss. In all cases, performance degrades to some extent. Overall, combining these three loss components provides a balanced objective that constrains velocity prediction, trajectory accuracy, and feasibility, resulting in the best performance.

**Figure 7:**
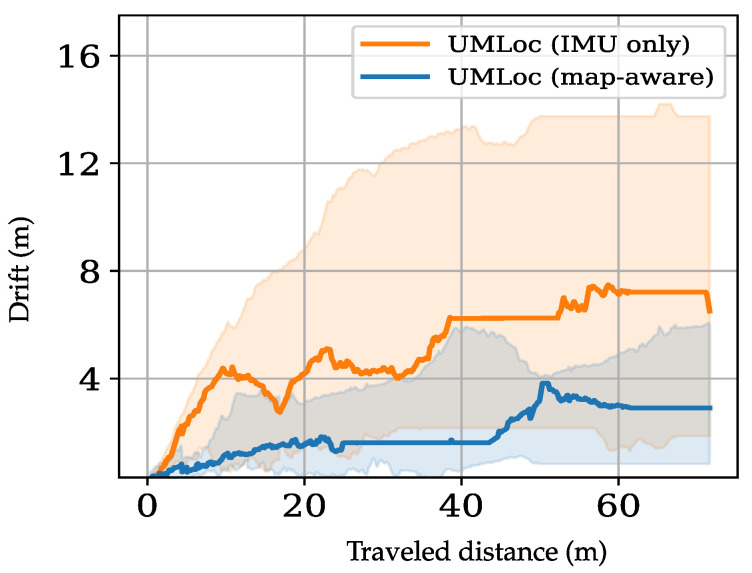
Drift error versus traveled distance for the map-aware model (UMLoc) and its IMU-only variant. Curves show the median drift across unseen test sequences. We computed the error over the average travel distance of 70m. The shaded area denotes the drift distribution over the testing trajectories. Incorporating map information (blue) constrains maximum drift growth to 5m throughout the testing trajectories versus 13m in the IMU-only model (orange), demonstrating the map’s effectiveness in long-distance indoor localization.

*(iii)* **Map conditioning.** To study the effect of map conditioning, we repeated the experiments using a *uniform feasibility map* instead of the true map. Figure 7 presents the drift-error analysis, defined as the position estimation error aggregated over trajectories and reported as a function of traveled distance up to a common horizon of 70m. Each trajectory’s drift-versus-distance curve is linearly interpolated onto a shared 70m grid before computing statistics.

The results reveal a substantial reduction in drift, validating the synergy between uncertainty bounds and spatial feasibility constraints. Notably, UMLoc with map conditioning consistently achieves lower cumulative drift than its IMU-only variant, effectively suppressing drift growth over distance. These findings underscore the critical role of map conditioning in improving localization accuracy, reducing positional uncertainty, and enhancing reliability during long-duration deployments.

#### 4.2.6. Generalization

Generalization is assessed along two dimensions: environment generalization and trajectory behavior generalization. Environment generalization examines whether a model trained on certain areas can transfer to unseen floor-plan geometries (e.g., different corridor topologies, room connectivity, and obstacle configurations). Trajectory behavior generalization evaluates whether the model can handle diverse pedestrian motion patterns within and across environments (e.g., changes in walking speed, stops and turns, long straight segments versus frequent direction changes), which directly affect IMU drift accumulation. Table 2 reports the evaluation of our model using held-out layouts and trajectories in a zero-shot setting. This protocol explicitly evaluates both cross-building (layout) transfer and cross-trajectory (behavior) transfer. Notably, in the cross-building evaluation, UMLoc maintains competitive accuracy across diverse layouts in our dataset In contrast, the model in [16] exhibits clear performance degradation under similar cross-building conditions, as discussed in the original reference.

In addition, we conduct a second generalization study comparing building-specific training with a universal model. For each building in our dataset, we trained an independent UMLoc model using only trajectories collected in that building and evaluated it on a held-out set of previously unseen trajectories from the same building, reporting the resulting ATE. We then averaged the ATE across all buildings. In parallel, we trained a single universal UMLoc model on the combined training data from multiple buildings and evaluated it on unseen trajectories using the same protocol. The comparison, summarized in Table 4, directly examined whether a single globally trained model can match the performance of specialized per-building models, thereby demonstrating generalization across both layout diversity and motion variability.

We attribute the strong generalizability of the single UMLoc model to three main factors: (i) the IMU encoder learns kinematic patterns that are agnostic primarily to the users and environments; (ii) device differences are mitigated through noise augmentation and the use of the quantile objective, which encourages calibrated and less over-confident predictions under data shifts; and (iii) the map-aware feasibility prior effectively constrains drift even in unfamiliar spaces, provided the floor plan is well-aligned with the global frame.

## 5. Conclusions

In this paper, we propose UMLoc, a novel localization framework that integrates an LSTM-based quantile-regression module with a map-conditioned CGAN to reduce drift and provide explicit prediction intervals. Through comprehensive experiments across our dataset and benchmark datasets, UMLoc consistently outperforms state-of-the-art approaches. The results demonstrate substantial improvements in accuracy, robustness and generalization capabilities. This work opens new avenues for researchers to explore more sophisticated integration techniques, extend the concept to various sensor modalities and address real-world localization challenges.

Future research directions include extending UMLoc to three-dimensional localization and addressing deployment challenges in real-world indoor navigation (i.e., shopping malls, airports, office buildings, hospitals and industrial facilities) and robotic applications.

## Figures and Tables

**Table 1:** Summary of dataset statistics.

Dataset	Travel Time (s)		Travel Distance (m)		Frequency
	Total	Mean ± Std	Total	Mean ± Std	(Hz)
RoNIN	82,332	552 ± 164	62,030	416 ± 154	200
RNIN	25,200	246 ± 318	27,820	92 ± 160	100
UMLoc	7488	125 ± 65	4050	67 ± 34	60

**Table 2:** Performance evaluation for UMLoc against three baseline models, RNIN-VIO and RoNIN LSTM (RoNIN-L)/TCN (RoNIN-T) on RoNIN, RNIN and our datasets. Improvement is calculated based on the best baseline model.

**RoNIN Dataset**			
**Model**	**FDE (%)**	**ATE (m)**	**RTE (m)**
RNIN	4.0	7.05	6.48
RoNIN-L	4.6	8.73	4.87
RoNIN-T	4.3	7.27	4.27
UMLoc (no map)	**3.5**	**6.58**	**3.93**
Improvement	12.50%	6.67%	7.96%
**RNIN Dataset**			
**Model**	**FDE (%)**	**ATE (m)**	**RTE (m)**
RNIN	**2.2**	1.59	2.57
RoNIN-L	8.0	2.66	3.93
RoNIN-T	5.2	2.72	3.90
UMLoc (no map)	3.4	**1.47**	**2.03**
Improvement	-	7.55%	21.01%
**Our Dataset**			
**Model**	**FDE (%)**	**ATE (m)**	**RTE (m)**
RNIN	17.2	4.65	4.61
RoNIN-L	23.2	4.14	4.94
RoNIN-T	19.2	3.66	4.62
UMLoc (no map)	12.1	2.33	3.23
Improvement	29.65%	36.34%	29.93%
UMLoc	**5.9**	**1.36**	**1.91**
Improvement	65.70%	62.84%	58.57%

**Table 3:** Ablation study of key components of UMLoc on our dataset.

Model	ATE (m)
UMLoc (ours)	1.36
No map conditioning	2.33
Without $L_{feas}$	1.67
No position term *p* in $L_{sup}$	1.63
No velocity term *v* in $L_{sup}$	2.98
Without attention	2.00

**Table 4:** Generalizability evaluation for UMLoc model using independent models for each building against one model for the combined buildings.

Model	ATE (m)
Building 1	1.12
Building 2	1.05
Building 3	0.89
Building 4	1.73
Building 5	0.95
Independent models average	1.15
All building model	1.36

## Data Availability

The dataset and codes used in this work are publicly available at https://github.com/m9alharbi/umloc.git (accessed on 22 February 2026).

## Appendix A. Post Quantile Calibration for RNIN

Besides the point estimate ${\hat{v}}_{t}\in R^{2}$, RNIN outputs a vector $cov_{t}\in R^{D}$ that parametrizes the diagonal predictive covariance. Following [17], each entry is interpreted as the logarithm of the per–dimension *standard deviation*,

(A1) $${\hat{\sigma }}_{t}\;=\;\left(\begin{array}{c} \exp(cov_{t,1})\\ \exp(cov_{t,2}) \end{array}\right).$$
